# Graph Convolutional Network for predicting secondary structure of RNA

**DOI:** 10.21203/rs.3.rs-3798842/v1

**Published:** 2024-02-23

**Authors:** Palawat Busaranuvong, Aukkawut Ammartayakun, Dmitry Korkin, Roya Khosravi-Far

**Affiliations:** 1 Department of Data Science, Worcester Polytechnic Institute, Worcester, 01609, Massachusetts, USA.; 2 Department of Computer Science, Worcester Polytechnic Institute, Worcester, 01609, Massachusetts, USA.; 3 InnoTech Precision Medicine, Boston, 02130, Massachusetts, USA.

**Keywords:** RNA Secondary Structure, Graph Convolutional Neural Network, Energy-based Model, RNAfold, SARS-CoV-2

## Abstract

The prediction of RNA secondary structures is essential for understanding its underlying principles and applications in diverse fields, including molecular diagnostics and RNA-based therapeutic strategies. However, the complexity of the search space presents a challenge. This work proposes a Graph Convolutional Network (GCNfold) for predicting the RNA secondary structure. GCNfold considers an RNA sequence as graph-structured data and predicts posterior base-pairing probabilities given the prior base-pairing probabilities, calculated using McCaskill’s partition function. The performance of GCNfold surpasses that of the state-of-the-art folding algorithms, as we have incorporated minimum free energy information into the richly parameterized network, enhancing its robustness in predicting non-homologous RNA secondary structures. A Symmetric Argmax Post-processing algorithm ensures that GCNfold formulates valid structures. To validate our algorithm, we applied it to the SARS-CoV-2 E gene and determined the secondary structure of the E-gene across the *Betacoronavirus* subgenera.

## 1 Introduction

RNAs play an essential role in biological processes, such as RNA translation, modification, and protein synthesis. Understanding the secondary structure of RNA is essential for the development of technologies in bioinformatics and medicine, including drug discovery, disease diagnostics, biosensing tools, and many other genomics applications. The secondary structure of RNA impacts its interaction with other cellular components. Accurate secondary structures can be determined through experimental assays, such as Nuclear Magnetic Resonance and X-ray Diffraction. However, these methods are often expensive, have resolution limits, and can be technically challenging.

Computational folding algorithms are alternative approaches to predict the secondary structure of RNA solely from its sequence. Energy-based models, such as RNAfold Lorenz et al (2011), RNAstructure Reuter and Mathews (2010), and UNAFold Markham and Zuker (2008), are commonly used for this purpose and are based on the idea that the most stable structure is the one that minimizes free energy (MFE) Tinoco and Bustamante (1999). Dynamic programming algorithms (DP) Zuker and Stiegler (1981); Trotta (2014) can be used to formulate the optimal MFE secondary structure, given a consistent estimator of the nearest-neighbor model Mathews et al (2004) to estimate the energy. This estimator is typically a linear combination of functions of substructures such as hairpins, internal loops, stems, etc. Zuker and Stiegler (1981). However, structure prediction accuracy using energy-based models is relatively low because the free energy parameters are experimentally determined in advance Sato et al (2021). Machine learning (ML)-based models, such as CONTRAfold Do et al (2006), Contextfold Zakov et al (2011), and MXfold Akiyama et al (2018), have been proposed to improve structure predictions by learning scoring parameters and finding structures with respect to them. CONTRAfold Do et al (2006) is a well-known algorithm that uses a stochastic context-free grammar rule to embed information on thermodynamic energy minimization and biological stability.

With the rise of AI technologies and the explosion of RNA sequence data, recent studies have used deep learning to predict secondary structures. SPOT-RNA Singh et al (2019) and E2Efold Chen et al (2020) both employ deep neural networks (DNN) for end-to-end RNA secondary structure prediction. These approaches can successfully predict pseudoknot structures, which traditional energy minimization techniques cannot. SPOT-RNA uses an ensemble of multiple DNN models to obtain the predicted structures. However, it may produce secondary structures that contain overlapping pairs (i.e., nucleotide pairs with more than one other base). E2Efold, on the other hand, comprises a deep score network and a post-processing network that introduces hard constraints over the DNN model to restrict the output space. However, recent studies Sato et al (2021); Fu et al (2022) have shown that E2Efold is prone to overfitting and is only effective for the specific sequence-wise RNA dataset on which it was initially trained. It is not capable of predicting RNAs from different families. To address this issue, MXfold2 Sato et al (2021) proposed a hybrid approach that combines DNN-based models and energy-based models. Integrating a folding score DNN and Turner’s nearest-neighbor free energy parameters helps prevent overfitting and improves the accuracy of secondary structure predictions. However, as with energy-based models, MXfold2 cannot predict complex pseudoknot structures.

Our work introduces a new graph convolutional network (GCN) model called GCNfold for predicting secondary structures of RNA. GCNfold is also an ensemble model, but unlike SPOT-RNA, which utilizes multiple DNN modules, GCNfold leverages graph neural networks to connect RNAfold’s base pairing probabilities with a DNN. To obtain stable RNA structures, the predictions from the deep score network are enforced by the following three constraints: only canonical base pairing with the inclusion of G-U pairing, no sharp loops, and each base pairing only once.

## 2 Results

GCNfold utilizes an ensemble model composed of the base pairing probability (BPP) matrix, calculated using McCaskill’s partition function algorithm McCaskill (1990), and an RNA one-hot encoding vector. The BPP matrix represents the structural connections between bases and can be conceptualized as a graph *G*(*V,E*). The core idea behind GCNfold is to formulate the prediction of RNA secondary structure as a binary classification problem, predicting whether each pair of nucleotides forms a complementary base pair according to the canonical and wobble base pairing rules or not. The model architecture and training process details are discussed in Section 3 of the Methods section. GCNfold undergoes training and evaluation in two benchmark datasets: the *Rivas Database* Rivas et al (2012) and the *bpRNA dataset* Danaee et al (2018). The inclusion of the *Rivas Database* allows an analysis of whether our richly parameterized model is prone to overfitting on a family-wise test dataset, while the *bpRNA dataset* aids the model in learning generalized RNA secondary structures, including pseudoknot pairings.

### 2.1 Performance Comparison on TestSet of Rivas Data

A comparative analysis of GCNfold with state-of-the-art algorithms for RNA secondary structure prediction, including MXfold2 Sato et al (2021), CONTRAfold Do et al (2006), RNAfold Zuker and Stiegler (1981), RNAstructure Reuter and Mathews (2010), ContextFold Zakov et al (2011), SimFold Andronescu et al (2007), and TORNADO Rivas et al (2012), was conducted using TestSetA and TestSetB for evaluation. In this experiment, MXfold2, ContextFold, and GCNfold were trained on TrainSetA. Table 1 presents the results, which shows that GCNfold achieves the highest F1 score on TestSetA. Furthermore, its F1 score on TestSetB, assessed on a family-wise basis, ranks second only to MXfold2’s F1 score. These findings highlight the effectiveness of hybrid approaches, such as MXfold2 and GCNfold, which combine free energy minimization and neural networks, in significantly improving the accuracy of RNA secondary structure prediction while mitigating overfitting concerns.

### 2.2 Performance Comparison on TS0 of bpRNA Data

**Fig. 2:**
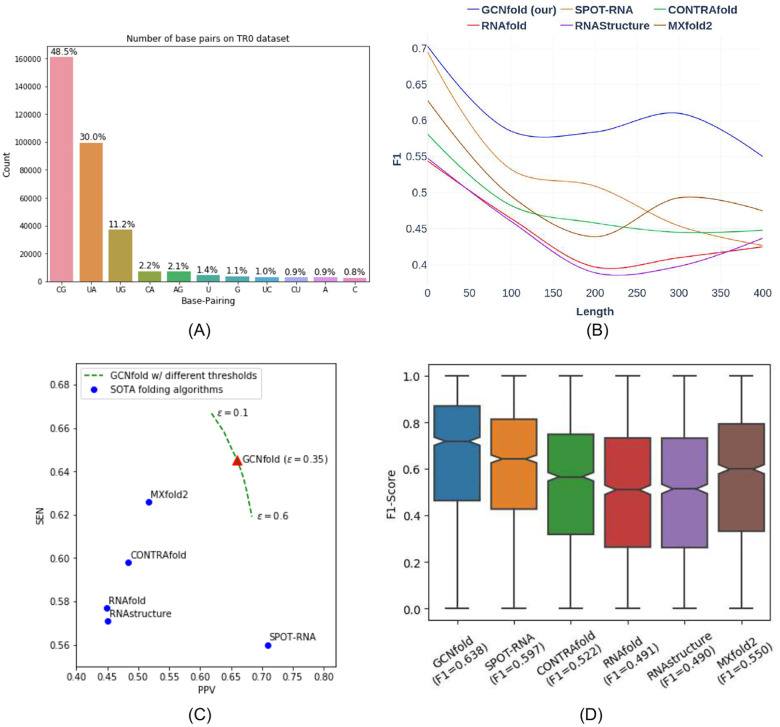
GCNfold achieves the highest F1 score on the bpRNA TS0 dataset. Exploratory and performance comparison of folding algorithms on the test set TS0 (A) shows the frequency of each type of base pairing within the bpRNA training set. (B) shows that GCNfold has a distinct distribution of F1 score distribution along the RNA length. Furthermore, the F1 distribution of GCNfold is relatively higher compared to other models. (C) shows the plot of the positive predictive value and sensitivity of the prediction of the model in the TS0 test set. *ε* represents the cutoff probability threshold of the model. The result shows that GCNfold has higher sensitivity than other models. (D) Box plot of the F1 score among the algorithms. GCNfold has the highest F1 score.

**Fig. 3:**
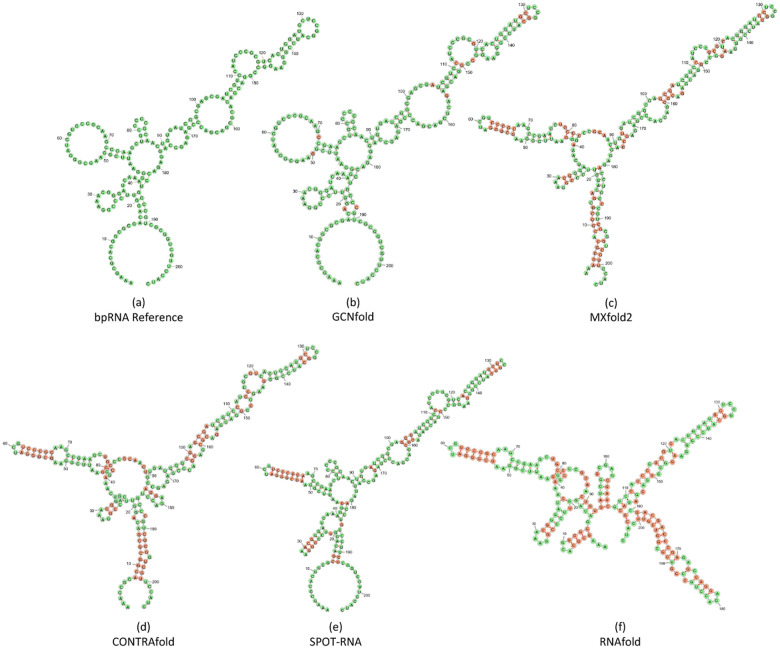
GCNfold prediction (b) yields the highest evaluation scores of F1=0.905, PPV=0.905, and SEN=0.905. Green bases represent nucleotides with the same structure types (pairing/non-pairing) as the bpRNA reference (a) while red bases represent incorrect predictions compared to the bpRNA reference. The scores of other models are as follows: (c) MXfold2: F1=0.481, PPV=0.403, SEN=0.595, (d) CONTRAfold: F1=0.558, PPV=0.468, SEN=0.690, (e) SPOT-RNA: F1=0.781, PPV=0.651, SEN=0.976, and (f) RNAfold: F1=0.257, PPV=0.209, SEN=0.333.

Next, we analyzed our model using a more comprehensive RNA dataset, which includes pseudoknot structures and various types of incorrect base pairings, in addition to canonical and wobble base pairings (see Figure 2(A)). Our model was trained on the TR0 dataset and validated on the VL0 dataset (please refer to Section 3 for more details on the bpRNA data). We compared GCNfold with state-of-the-art (SOTA) algorithms for RNA secondary structure predictions in the TS0 dataset. The results, presented in Figure 2(D), reveal that GCNfold achieves the highest F1 score of 0.638 in the bpRNA dataset. Although GCNfold’s F1 score is approximately 4% higher than SPOT-RNA’s score, it strikes a better balance between sensitivity (SEN) and positive predictive value (PPV) scores. This indicates that GCNfold is a more reliable algorithm for predicting true base pairings. Additionally, it should be noted that the predictions from SPOTRNA may not always adhere to the three hard constraints necessary to form valid secondary structures. An example of these constraint violations can be observed in Figure 3(e), where (1) there are types of pairings in addition to (A-U), (C-G) and (G-U), and (2) the structure of the hairpin loop (between the 29^*th*^-30^*th*^ bases) is invalid because there must be at least three bases inside a stem.

The GCNfold scores significantly exceed the RNAfold scores, serving as our model’s edge features. This implies that GCNfold enhances the predictive capabilities of RNAfold by assimilating its underlying pairing distribution through a graph network, thus formulating a more precise posterior pairing distribution, i.e., predicted base pairing probabilities. We also analyzed our model’s predictive performance across various RNA length intervals. In particular, for shorter RNA sequences (*L* ≤ 100 bases), all models produce higher evaluation scores than longer RNA sequences. Intriguingly, our models maintain an F1 score of approximately 0.58–0.61 for RNA sequences with lengths ranging from 100 to 400 bases, slightly lower than GCNfold’s average F1 score of 0.638.

### 2.3 Analysis of GCNfold Predictions on Short Segments of the SARS-CoV-2 Viral Genome

Experimental approaches to obtain a highly accurate secondary structure of RNA, especially for long RNA genomes, are time-consuming Wu et al (2020). This can become a substantial bottleneck in clinical applications, specifically when studying genomes of RNA viruses, such as the recently emerging SARS-CoV-2 (Refseq accession number NC 045512.2), a single-stranded betacoronavirus with a genome of approximately 30 kilobases. It is still challenging for computational folding algorithms to predict the structure of a long genome at once due to structural complexity and computational demands. In this experiment, *GCNfold* is employed to predict the secondary structures of small genes or subsections of the SARS-CoV-2 genome, such as the 5’ untranslated region (UTR), heptapeptide repeat regions (HR) of the spike (S) gene, and the envelope (E) gene. The validation of our model predictions is compared with the available SARS-CoV-2 structure from Lan et al (2022) based on DMS mutational profiling with sequencing (DMS-MaPseq) Zubradt et al (2017).

**Fig. 4:**
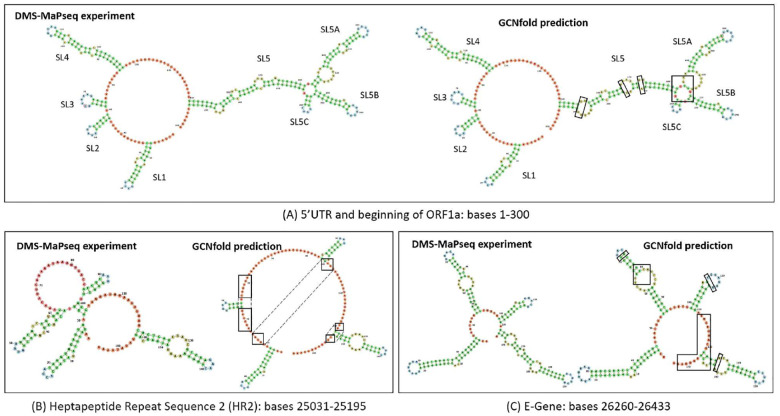
Generated secondary structures of SARS-CoV-2 sub-genomes by GCN-fold are homogeneous to those formulated by DMS-MaPseq. (A) Consensus secondary structure predictions of 5’UTR and beginning of ORF1a structure, (B) HR2 region, and (C) E-gene by GCNfold and DMS-MaPseq, respectively. Black boxes indicate the regions where the GCNfold structure predictions differ from the DMS-MaPseq determinations.

The GCNfold prediction demonstrates that our algorithm accurately predicts most of the 5’UTR and the beginning of the ORF1a structure, with an F1 score of 0.953, a PPV of 0.988, and a SEN of 0.921 compared to the DMS-MaPseq reference structure (Figure 4(A)). In line with a previous study Zubradt et al (2017), GCNfold perfectly predicted four stem loops (SL1–4) within the 5’ UTR, with minor differences found in the SL5 region, where our model formulated a larger multibranch loop that included SL5A, SL5B and SL5C. The prediction scores for other folding algorithms are RNAfold (F1=0.924, PPV=0.885, SEN=0.966) and MXfold2 (F1=0.960, PPV=0.955, SEN=0.966). Therefore, our model not only enhances the prediction accuracy of RNAfold but also achieves a performance comparable to MXfold2.

We then applied our method to the gene that encodes the SARS-CoV-2 Spike protein, which plays an essential role in the interaction with potential host cells by binding its receptor-binding domain to the host receptor, causing an infection Huang et al (2020); Zhou and Zhao (2020). GCNfold achieved an F1-score of 0.845, a PPV of 0.968, and a SEN of 0.750 for predicting the secondary structure of HR2, a part of the S2 subunit of the spike protein that plays a role in the virus’s membrane fusion with the host Lu et al (2015). Interestingly, the energy-based model (e.g., RNAfold) completely failed to predict the structure of this particular gene (F1=0.118, PPV=0.111, SEN=0.125). MXfold2, whose post-processing heavily relies on Zuker’s algorithm, could not formulate an accurate structure (F1=0.500, PPV=0.563, SEN=0.450). Despite the high evaluation scores, GCNfold’s prediction missed the interactions between bases marked by the black boxes.

Lastly, we predicted the folding structure of the E-gene (Figure 4(C)). We observed that the predictions of the first two stem loops by GCNfold are almost identical to the DMS-MaPseq structure. In this region, MXfold2 performed the best in predicting its secondary structure. The scores for the folding algorithms are as follows: GCNfold (F1=0.855, PPV=0.917, SEN=0.800), MXfold2 (F1=0.924, PPV=0.961, SEN=0.891), RNAfold (F1=0.815, PPV=0.830, SEN=0.800).

### 2.4 Comparative analysis of secondary structures for GCNfold Predictions of E-gene across *Betacoronavirus* Subgenera

This study aims to analyze the RNA secondary structure of the envelope protein gene (E gene) across various subgenera within the Betacoronavirus genus. These subgenera include SARS-CoV-1 (Sarbecovirus, lineage B) Thiel et al (2003), RaTG13 (Sarbecovirus, lineage B) Zhou et al (2020), and MERS-CoV (Merbecovirus, lineage C) van Boheemen et al (2012). The selection of the E gene stems from its presence in all Betacoronavirus subgenera and its relatively short genome, rendering it well-suited for comparative analysis. There is a notable absence of experimental data regarding the annotated RNA secondary structure within these subgenera. Consequently, this study aims to shed light on these structural aspects and underscore the effectiveness of the GCNfold model in making these inferences.

**Fig. 5:**
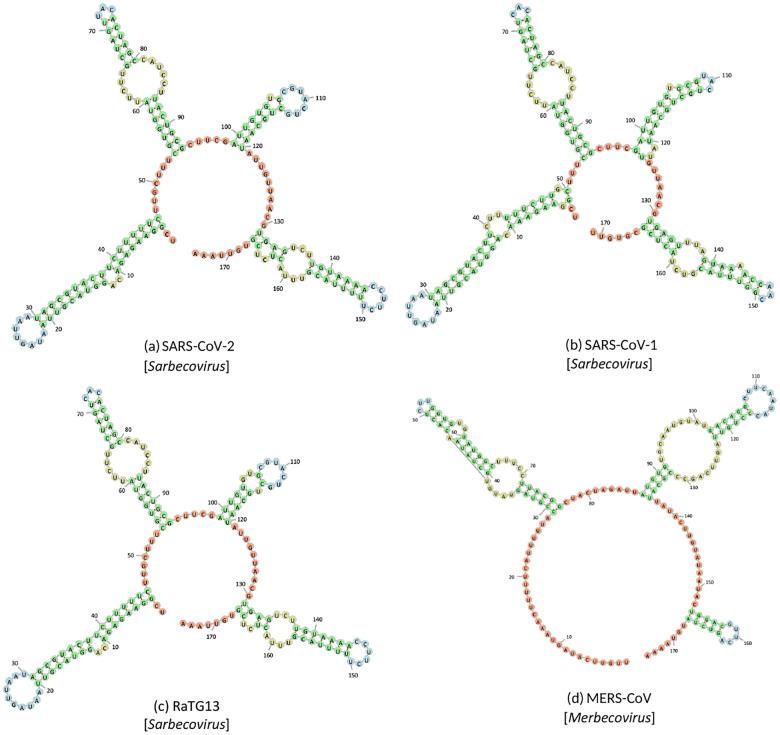
Formulated secondary structures of E genes of *Betacoronavirus* by GCN-fold shows the similarity within their subgenus. Where (a), (b), and (c) are the structures of SARS-CoV-2 (NC 045512.2), SARS-CoV-1 (AY291315.1), and RaTG13 (MN996532.2) that belong to the *Sarbecovirus* linkage. And (d) is the structure MERS-CoV (NC 019843.3), which belongs to the *Merbecovirus* linkage.

Based on the GCNfold predictions (Figure 5), the anticipated structures of the E genes within the Sarbecovirus subgenus (Figure 5(a), (b), and (c)) exhibit striking similarities. Notably, whether non-silent or silent mutations, these variations do not significantly impact the overall secondary structure. In contrast, the projected structure of the E gene from the Merbecovirus subgenus (Figure 5(d)) markedly deviates from that of the Sarbecovirus subgenus due to substantial disparities in their nucleotide sequences, potentially influencing protein synthesis. This shows the GCNfold model’s ability to accurately predict the RNA secondary structure of the E gene across diverse subgenera. Furthermore, in the process of secondary structure prediction, this algorithm identifies regions within the E gene that can serve as a basis for designing subgenus-specific primers for molecular diagnostics. Alternatively, these regions can be harnessed to develop target-specific or target-agnostic antisense primers for therapeutic purposes.

## 3 Methods

### 3.1 Data Descriptions

We employ two benchmark datasets to train our model and assess its performance in comparison to other folding algorithms:

- *Rivas Database* Rivas et al (2012): This dataset comprises TrainSetA, TestSetA, and TestSetB. TrainSetA and TestSetA contain sequences with less than 70% sequence identity compared to the data in TestSetB, ensuring non-homologous sequences. Additionally, the dataset excludes pseudoknot secondary structures Sato et al (2021). In this scenario, our model was trained on TrainSetA and evaluated on TestSetA (sequence-wise) and TestSetB (family-wise). Table 2 summarizes the statistics related to this dataset.
- (*ii*)*bpRNA-1m dataset* Danaee et al (2018): This dataset’s secondary structures are based on experimental data from various studies. To mitigate the risk of overfitting due to dataset redundancy and sequence similarity, we followed SPOT-RNA’s protocol Singh et al (2019). This protocol employed an 80% sequence identity cut-off, computed using the CD-HIT-EST program Fu et al (2012). The cut-off dataset is then randomly split into training (TR0), validation (VL0), and testing (TS0) sets, as indicated in Table 2. Exploratory data analysis on the TR0 dataset reveals that approximately 78.5% of base-pairing occurrences are canonical (G-C & A-U), 11.2% are non-canonical Wobble pairings (U-G), and the remaining 10.3% consist of other non-canonical pairings.

### 3.2 Modeling

**Fig. 6:**
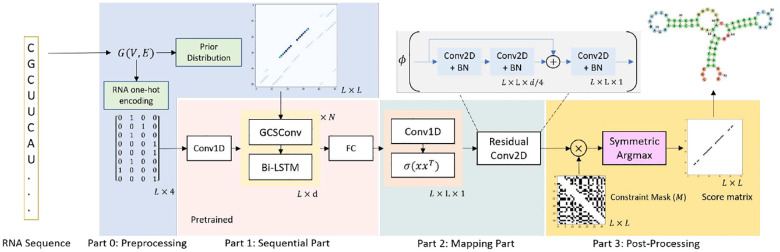
Deep neural architecture of GCNfold The input of the model is a set of graph G such that for each graph *G*(*V,E*), *V* is the encoded information of RNA sequences which is *L* × 4 matrix in which each row representing the nucleotide for each base. The edge *E* will be the pairing probability matrix from passing RNA sequences through RNAfold. That input then gets processed by the graph convolutional layer (GCSConv) and bi-directional LSTM for generating the embedding. This part is pre-trained before training the whole model. Then, the embedding is converted back to the matrix and passed through the series of convolutional layers with a skip connection. The output of convolutional layers is post-processed by applying an element-wise product with a constraint mask. Last, the symmetric argmax algorithm is applied to formulate the score matrix, which is the binary representation of RNA secondary structure.

Figure 6 presents the architecture of our deep neural network denoted as *F*_*θ*_(*G*), designed for calculating base pairing probability scores. Here, *θ* represents learnable parameters, and *G* signifies the input graph. Our model operates as an ensemble, incorporating the base pairing probability (BPP) matrix derived from RNAfold Lorenz et al (2011) and the RNA embedding vector. The BPP encodes structural connections between bases, forming a graph *G*(*V,E*). We employ a graph convolutional network (GCN) to harness this information. It takes the *L* × *d* dimensional sequence embedding of an RNA sequence with length *L* as node features (*X*) and the *L* × *L* prior BPP as a quasi-adjacency matrix (${\hat{A}}_{C}$) of graph *G*. The propagation process in a general GCN layer *f*(*X,A*) is defined as follows:

(1) $$f(X,A)=\sigma \left({\tilde{D}}^{-1/2}\tilde{A}{\tilde{D}}^{-1/2}XW+b\right)$$

Here, *Ã* = *A*+*I*, where *I* represents added self-loops, and ${\tilde{D}}^{-1/2}$ stands for the inverse of the degree matrix corresponding to *Ã*. The parameters *W* and *b* are trainable weights and biases for the function *f*(*X,A*). This process approximates the convolution operator within a graph Defferrard et al (2016); Kipf and Welling (2016) (for further details, see Kipf and Welling (2016)). In our work, we have enhanced *f*(*X,A*) by introducing a trainable skip connection. Notably, the RNAfold BPP already generates a stochastic quasi-adjacency matrix *Â*_*R*_. Consequently, there’s no need to include the self-loops *I* as they are irrelevant for RNA secondary structures (i.e., *a*_*ij*_ = 0 when *i* = *j*). Additionally, we can omit ${\tilde{D}}^{-1/2}$ since there’s no requirement to renormalize the probability matrix. Thus, our modified *f*(*X,Â*_*R*_) (also referred to as the *GCSConv* layer) can be expressed as follows:

(2) $$f\left(X,{\hat{A}}_{R}\right)=\sigma \left({\hat{A}}_{R}XW_{1}+XW_{2}+b\right)$$

In this equation, *W*1, *W*2, and *b* represent parameters that must be trained, and *σ*(·) denotes the nonlinear activation function. Specifically, we employ GeLU Hendrycks and Gimpel (2020) as the activation function in this model. Figure 6 depicts our model divided into three stages.

#### 3.2.1 Stage 1: Sequential

In this stage, we start by inputting the graph-structured data into the GCSConv block, followed by the bidirectional long short-term memory (Bi-LSTM) block. To maintain computational efficiency, each direction of the Bi-LSTM network has a total of *d/*2 hidden units. The combination of *GCSConv+Bi-LSTM* blocks is repeated a total of *N* times. The resulting output feature map is then forwarded to a fully connected layer (FC) responsible for learning and predicting the probability matrix. This matrix has dimensions *L* × (*p* + 1), where *L* represents the length of the sequence, and *p* signifies the number of possible dot-bracket notations. The neural network architecture in this stage can be viewed as a sequence-to-sequence model, where the input sequence is transformed into a corresponding sequence of dot-bracket notations.

#### 3.2.2 Stage 2: Mapping

In this stage, we convert the sequential information acquired from Stage 1 into a binary score matrix with dimensions *L* × *L* using a convolutional network. Initially, a *Conv1D* layer generates a matrix *x* sized *L* × *d/*2. After that a matrix multiplication between *x* and its transpose *x*^*T*^ results in a matrix of size *L* × *L*. Subsequently, a non-linear activation function *σ*(·) is applied to this result. The output is passed through to the *Residual Conv2D* block, in which the network detail is illustrated in Figure 6. The sigmoid activation *ϕ*(·) is then used to compute the output pairing probability matrix of *F*_*θ*_(*G*)

#### 3.2.3 Stage 3: Post-Processing

As previously mentioned, biologically, we assume that RNA secondary structures must satisfy the following constraints Chen et al (2020); Steeg (1993):

1. Only allows Watson-Crick and Wobble pairing types, denoted as *λ* = {(*A,U*),(*U,A*),(*C,G*),(*G,C*),(*U,G*),(*G,U*)}
2. Prohibiting sharp loops in the secondary structure means loops must not consist of less than three nucleotides.
3. Restricting each base to be paired with at most one other base (i.e., each row and column of *Â* must have at most one non-zero element).

We implement constraints (i) and (ii) using a symmetric constraint matrix *M*, where *M* ∈ 0,1^*L*×*L*^. Here, *x*_*i*_ represents a base at the *i*^*th*^ position of the RNA sequence *x* = (*x*_1_, …*, x*_*L*_). The matrix *M* is defined as follows: *M*_*ij*_ = 1 if (*x*_*i*_*, x*_*j*_) ∈ *λ* and i-j ≥ 4; otherwise, *M*_*ij*_ = 0 (as illustrated in Figure 6). To ensure that the output matrix of *F*_*θ*_(*G*) adheres to constraints (i) and (ii), we perform element-wise multiplication between *F*_*θ*_(*G*) and *M*, denoted as *Â* = *F*_*θ*_(*G*) ⊗ *M*. This operation simplifies the search space of GCNfold, allowing the training process to converge significantly faster than training the model without including matrix *M*.


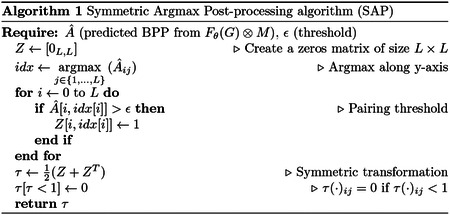


However, the prediction matrix *Â* may not always satisfy the constraint (iii). To address this issue, we propose a *Symmetric Argmax Post-processing* (SAP) technique inspired by Booy et al (2021). The SAP algorithm, outlined in Algorithm 1, is straightforward. Given a predicted probability matrix *Â*, we perform an argmax operation along the y-axis to identify the position *i* of the maximum value in the *j*^*th*^ column. Next, we introduce a probability threshold (*ϵ*) set at 0.35 as a cutoff to determine which *Â*_*ij*_ values can form pairings. Because the score matrix *Â* does not guarantee symmetric pairings, we define a transformation *τ* on {0, 1}^*L*×*L*^ as $\tau (\hat{A})=\frac{1}{2}\left(\hat{A}+{\hat{A}}^{T}\right)$, where *τ*(*Â*)_*ij*_ = 0 if *τ*(*Â*)_*ij*_ < 1. As a result, the output of GCNfold is represented by the predicted RNA secondary structure in the form of a matrix *τ*(*Â*) ∈ {0, 1}^*L*×*L*^. This matrix is symmetric and complies with all three sets of constraints (i), (ii), and (iii). The *ϵ* value of 0.35 is optimized on the VL0 dataset (selected values: 0.1, 0.15, 0.2, 0.25, 0.3, 0.35, 0.4, 0.45, 0.5, and 0.6).

### 3.3 Training Procedure

We leverage the idea of transfer learning to train GCNfold. Specifically, we divide the training process into two steps: (1) Training GCNfold during Stage 1 to formulate dot-bracket notations and (2) Connecting the neural network from Stage 1 with other parts of the architecture, and fine-tune the whole network to predict the base pairing probability matrix (i.e., the score matrix). The training process is described below.

Step 1: (Pre-training Step). As described in Stage 1 (3.2.1), given RNA graph data *G*(*V,E*) as input, we train the sequence-to-sequence model to predict secondary structures in the form of dot-bracket sequences. We use the training dataset (TR0) for training and assessing the model on the validation dataset (VL0). In particular, we use categorical cross-entropy loss and Adam optimizer for training the model. The hyperparameters *d* and *N* are also optimized in this step (i.e., selecting the hyperparameters’ combination that returns the lowest loss after training for 60 epochs).

Step 2: After finishing the initial training of Step 1, we connect the model defined in Stage 1 with the other sections (Figure 6). The neural network weights from Stage 1 are initialized by the trained parameters of the sequence-to-sequence model in Step 1. Now, we predict the base pairing probability matrix (BPP) due to its dimension and sparsity compared to the dot-bracket sequence. Since BPP is a sparse matrix (most entries are 0), we use weighted binary cross-entropy loss with a positive sample weight of 100 to handle imbalanced label predictions during training. The Adam optimizer and a reduced learning rate on the plateau are utilized when a metric stops improving for more than 5 epochs.

#### 3.3.1 Performance Measure

The metrics commonly used for this task are Sensitivity (*SEN*), Positive Predictive Value (*PPV*), and *F*1 score. *SEN* measures the ability to predict the positive base pairs, while *PPV* measures the ability not to fold false positive base pairs Wang et al (2019). Finally, the *F*1 score is the harmonic mean of *SEN* and *PPV*, a balanced metric between *PPV* and *SEN*. The equations of our metrics are as follows:

(3) $$SEN=\frac{TP}{TP+FP},\;PPV=\frac{TP}{TP+TN}\text{, and }F1=\frac{2\times SEN\times PPV}{SEN+PPV}$$

Here, *TP* is the number of correctly predicted pairs, *FP* is the number of wrong-predicted pairs, and *TN* is the number of correctly predicted unpaired bases.

## 4 Conclusion

This study introduces the GCNfold model, a graph convolutional neural network designed to predict RNA secondary structures from RNA sequences. We also present a symmetric argmax post-processing algorithm with linear time complexity integrated into the model. This algorithm enforces the secondary structure constraints and ensures all output predictions are valid. In our initial experiment, we demonstrate that GCNfold outperforms other folding algorithms, particularly on the testing set (TS0) of the bpRNA dataset, where the RNA sequences in the testing set exhibit structural similarity to those in the training set. Notably, GCNfold shows an ability to recognize certain pseudoknot structures.

Concerns regarding overfitting have been mentioned in previous research, particularly in rich-parameterized models Rivas et al (2012); Sato et al (2021). To mitigate this issue, GCNfold leverages base pairing probabilities obtained from RNAfold partition as a known prior distribution of RNA graph data. It then embeds RNA structural data through multiple graph convolutional layers and optimizes DNN parameters. As a result, our experiments reveal that GCNfold achieves the best F1 score when applied to homologous sequences from TestSetA. Furthermore, even for non-homologous sequences from TestSetB, its F1-score is second only to MXfold2, with a difference of approximately 1%. This outcome suggests that incorporating prior information related to free energy and graph structural data into deep neural networks can significantly enhance prediction accuracy and model robustness.

The experiment on SARS-CoV-2 genomes also demonstrates the utility of folding algorithms for newly discovered RNA. The results suggest that the predicted structures from the learning models leveraging thermodynamic energy knowledge (e.g., GCNfold and MXfold2) could formulate structures that closer resemble the structures obtained from the DMS-MaPseq experiment than a traditional energy-based model (e.g., RNAfold).

Due to limited training data for RNA secondary structure prediction, accuracy remains constrained. One potential improvement path involves using unsupervised learning algorithms to enhance our understanding of RNA sequences. Despite current limitations, GCNfold can still be valuable for RNA structure prediction, especially when combined with expert knowledge like primer-nucleotide design or identifying RNA-exposed regions. In our study, we employed a basic graph convolutional layer Kipf and Welling (2016). Future research could explore alternative convolutional layer designs Bianchi et al (2021); Thekumparampil et al (2018); Xu et al (2019) used in graph neural networks (GNNs) to potentially boost RNA secondary structure prediction accuracy. Additionally, adapting algorithms like sliding windows Agarwal et al (2019); Chen et al (2006) or leveraging RNA’s physical properties, as demonstrated in AlphaFold Senior et al (2020, 2019), are avenues worth exploring for enhanced performance given the long RNA sequences.

## Figures and Tables

**Fig. 1:**
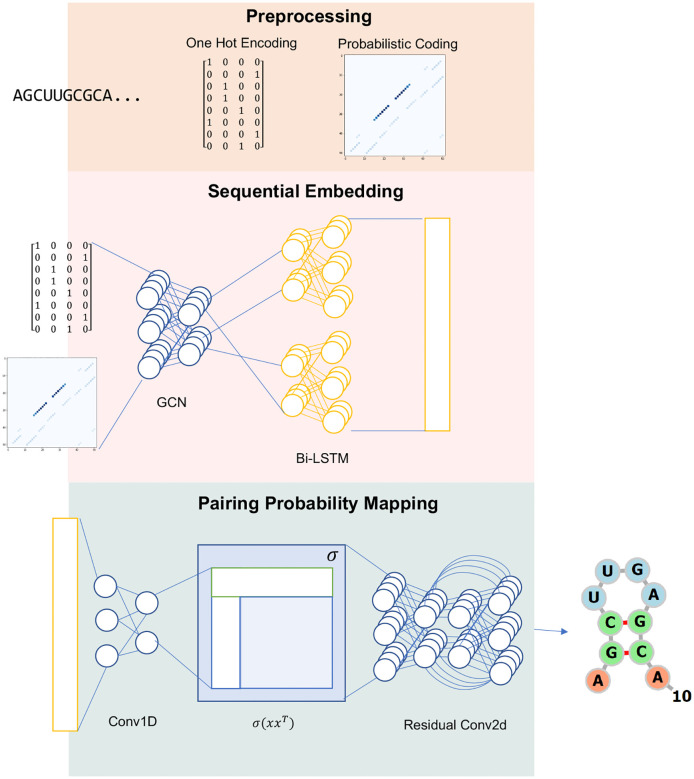
Workflow of the modeling and inference The prediction process began with the preprocessing of the RNA Sequence. The one hot encoding from the sequence itself and the probabilistic encoding from Vienna RNAfold Lorenz et al (2011) were used as input for the sequential embedding part, which consisted of a graph convolutional network (GCN) and bidirectional LSTM (Bi-LSTM). The Bi-LSTM output was then flattened and inserted into the pairing probability mapping, which expanded the dimension of a vector into a matrix with a one-dimensional convolution network and the shape-preserved nonlinear transformation *σ* of the inner product between itself and its transpose. The output then passed through the residual networks and underwent post-processing to yield the RNA secondary structure.

**Table 1:** GCNfold yields the same performance as MXfold2 on the TestSetA and non-homologous TestSetB. Where TestSetA shares less than 70% sequence identity with TestSetB.

	TestSetA (sequence-wise)			TestSetB (family-		wise)
	PPV	SEN	F1	PPV	SEN	F1
GCNfold (ours)	**0.801**	0.773	**0.780**	**0.589**	0.603	0.587
MXfold2	0.745	**0.778**	0.761	0.571	**0.650**	**0.601**
CONTRAfold	0.671	0.705	0.682	0.543	0.629	0.575
RNAfold	0.626	0.668	0.642	0.498	0.606	0.540
RNAstructure	0.622	0.650	0.631	0.475	0.584	0.518
ContextFold	0.768	0.750	0.759	0.485	0.534	0.502
SimFold	0.616	0.643	0.629	0.512	0.611	0.551
TORNADO	0.738	0.754	0.746	0.528	0.594	0.552

**Table 2:** The summary of datasets used in our experiments.

Dataset		#sequences	Length
bpRNA-1m (cut-off)	TR0	10,841	33–498
	VL0	1,300	33–497
	TS0	1,305	22–499
Rivas Data	TrainSetA	3,166	10–734
	TestSetA	592	10–768
	TestSetB	430	27–244

**Fig. A1:**
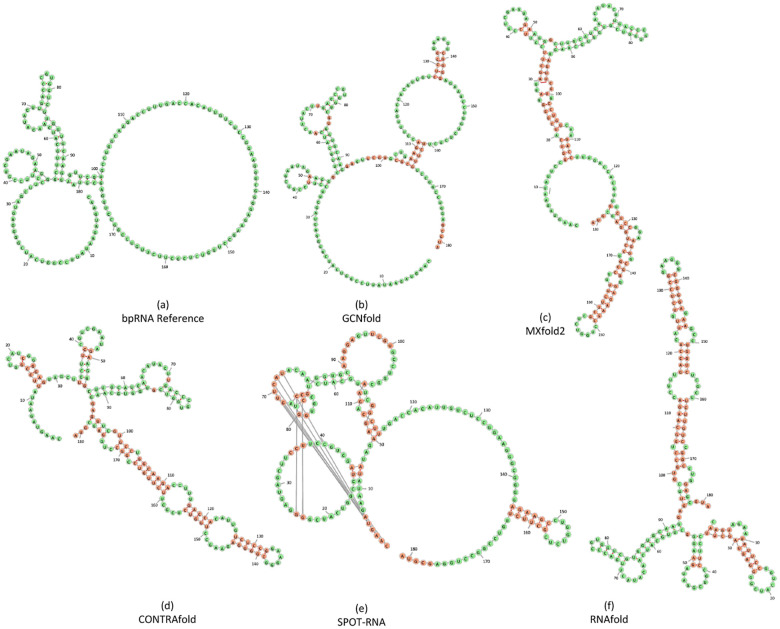
Comparison of predicted structures of bpRNA-RFAM-6232: Green represents the correct prediction, and red indicates the incorrect prediction in that position compared to (a) bpRNA reference. The predictions are from (b) GCNfold (F1=0.595, PPV=0.583, SEN=0.609), (c) MXfold2 (F1=0.453, PPV=0.323, SEN=0.304), (d) CONTRAfold (F1=0.444, PPV=0.326, SEN=0.696), (e) SPOT-RNA (F1=0.264, PPV=0.233, SEN=0.400), and (f) RNAfold (F1=0.430, PPV=0.303, SEN=0.739).

**Fig. B2:**
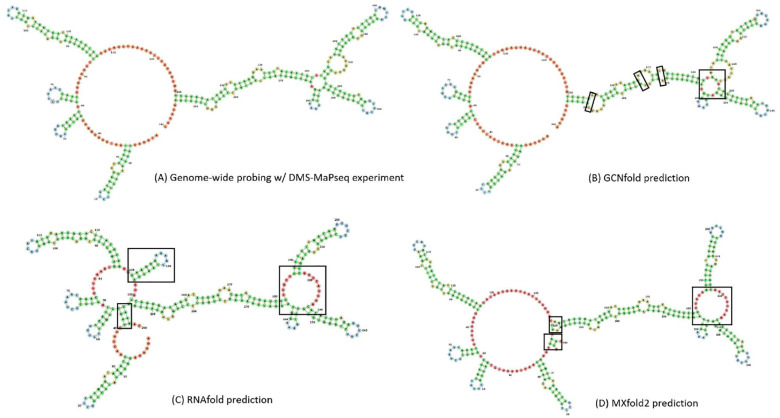
Secondary structure predictions of 5’UTR and beginning of ORF1a structure by (A) DMS-MaPseq (reference), (B) GCNfold, (C) RNAfold, and (D) MXfold2

**Fig. B3:**
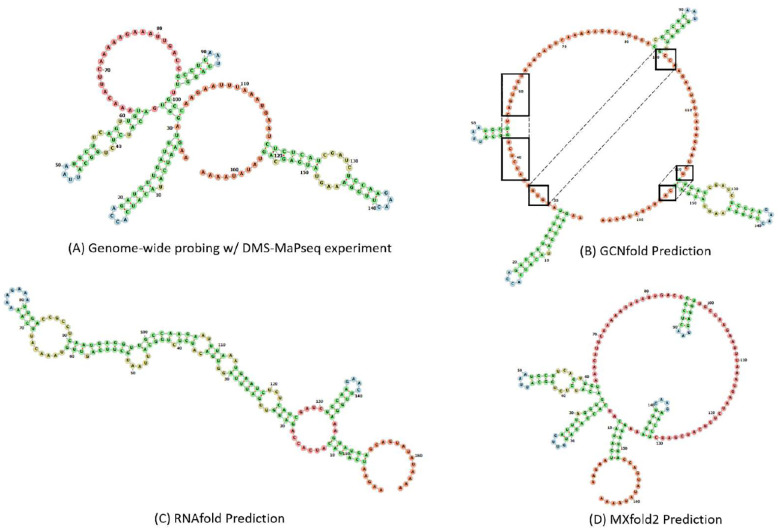
Secondary structure predictions of Heptapeptide Repeat Sequence 2 (HR2) by (A) DMS-MaPseq (reference), (B) GCNfold, (C) RNAfold, and (D) MXfold2

**Fig. B4:**
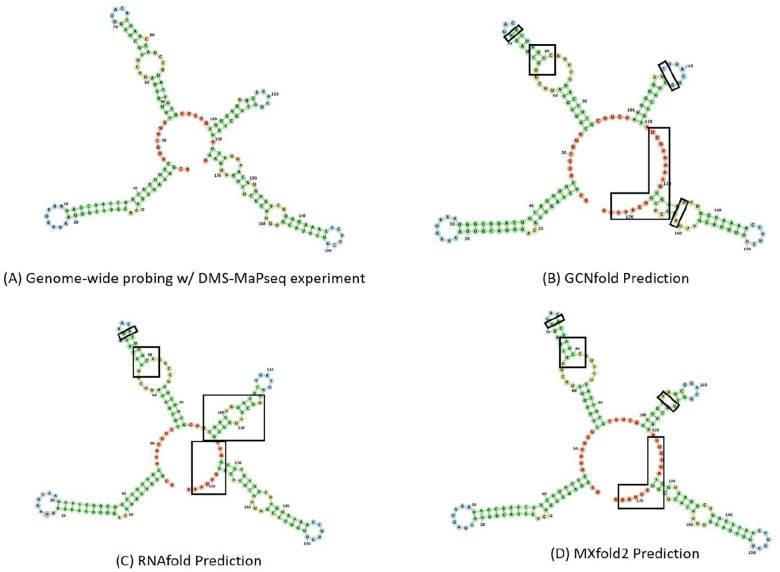
Secondary structure predictions of envelope (E) gene by (A) DMS-MaPseq (reference), (B) GCNfold, (C) RNAfold, and (D) MXfold2

**Fig. C5:**
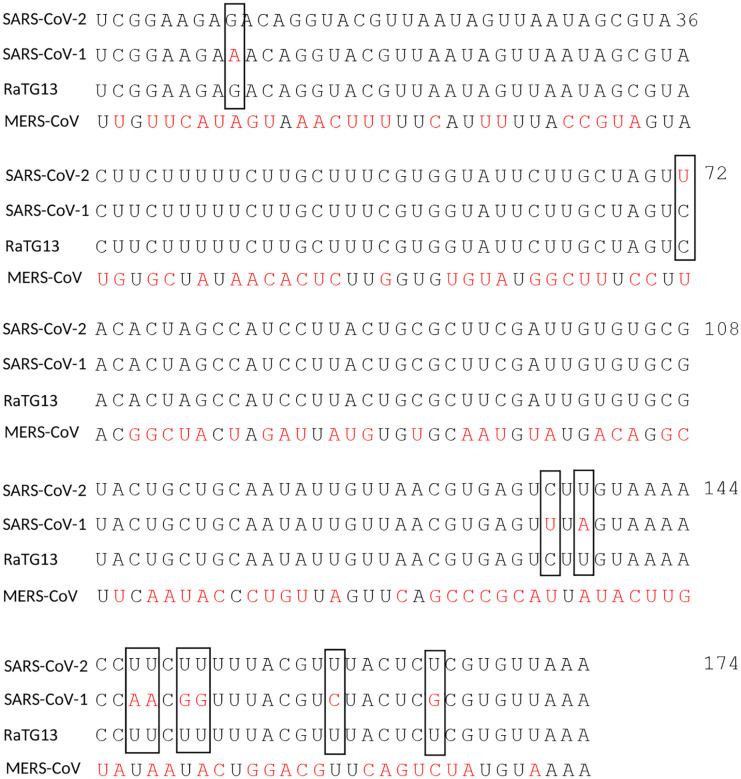
Comparison of E-gene RNA sequences among some of Coronavirus. The rectangle indicates the mutation position and the nucleotide highlighted with red color indicates the difference in *Sabercovirus*. In which, SARS-CoV-2 and RaTG13 have only one difference in a nucleotide at the 72th position.

## Appendix A An example of RNA structure where none of the models can predict correctly

A1 shows a case in which neither model can predict secondary structures that are similar to the bpRNA reference.

## Appendix B SARS-CoV-2 structure prediction by each folding algorithm

The scores of folding algorithms on this region compared with the DMS-MaPseq reference are as follows: GCNfold (F1=0.953, PPV=0.988, SEN=0.921), RNAfold (F1=0.924, PPV=0.885, SEN=0.966), and MXfold2 (F1=0.960, PPV=0.955, SEN=0.966).

The scores of folding algorithms on this region compared with the DMS-MaPseq reference are as follows: GCNfold (F1=0.845, PPV=0.968, SEN=0.750), RNAfold (F1=0.118, PPV=0.111, SEN=0.125), and MXfold2 (F1=0.500, PPV=0.563, SEN=0.450).

The scores of folding algorithms on this region compared with the DMS-MaPseq reference are as follows: GCNfold (F1=0.854, PPV=0.917, SEN=0.800), RNAfold (F1=0.815, PPV=0.830, SEN=0.800), and MXfold2 (F1=0.924, PPV=0.961, SEN=0.891).
